# Hypertension in Jordan: Prevalence, Awareness, Control, and Its Associated Factors

**DOI:** 10.1155/2019/3210617

**Published:** 2019-05-02

**Authors:** Yousef Khader, Anwar Batieha, Hashem Jaddou, Sukaina I. Rawashdeh, Mohammed El-Khateeb, Dana Hyassat, Albaraa Khader, Kamel Ajlouni

**Affiliations:** ^1^Department of Public Health, Jordan University of Science and Technology, P.O. Box 3030, Irbid 22110, Jordan; ^2^Department of Public Health, Jordan University of Science and Technology, Irbid, Jordan; ^3^Department of Internal Medicine, Jordan University of Science and Technology, Irbid, Jordan; ^4^The National Center for Diabetes, Endocrinology, and Genetics, Jordan University, Jordan; ^5^Faculty of Medicine, Jordan University of Science and Technology, Irbid, Jordan

## Abstract

**Objectives:**

Determine the prevalence, awareness, and control rates of hypertension and their associated factors among Jordanian adults.

**Methods:**

A multistage sampling technique was used to select a nationally representative sample of adults from the population of Jordan. Trained interviewers collected data using a comprehensive structured questionnaire, measured anthropometric parameters, and collected blood samples.

**Results:**

This study included a total of 1193 men and 2863 women aged ranged from 18 to 90 year with a mean (SD) of 43.8 (14.2) year. The age-standardized prevalence was 33.8% among men and 29.4% among women. Of those with hypertnsion, 57.7% of men and 62.5% of women were aware of hypertension. Only 30.7% of men and 35.1% of women who were on antihypertensive medications had their blood pressure controlled. From 2009 to 2017, there was nonsignificant decrease in hypertension prevalence of 2.7% among men and 1.1% among women. However, the rate of hypertension awareness increased significantly among men and among women.

**Discussion:**

Almost one-third of Jordanian adults had hypertension. Interventions that target modifiable risk factors of hypertension, might decrease blood pressure, and even prevent the development of hypertension should be implemnted.

## 1. Introduction

Hypertension is a modifiable risk factor for cardiovascular and cerebrovascular diseases worldwide [1]. The burden of hypertension is very high because of its high prevalence and its associated mortality and morbidity [2]. One study showed that the prevalence of hypertension is expected to increase by 7.2% from 2013 estimates by 2030 [3]. The complications of hypertension account for 9.4 million deaths worldwide every year and it is estimated that up to 1.58 billion adults will suffer from complications of hypertension by 2025, worldwide [4, 5]. Hypertensive heart disease was the fourth-highest ranked cardiovascular disease cause for DALYs in 2015 globally [6].

About 30% of adults in Arab countries were estimated to have hypertension [7]. In a follow-up study in Jordan comparing hypertension prevalence from 1994 to 2009, the prevalence of hypertension increased from 29.4% to 32.3% [8]. Although screening, early detection, and control of hypertension are associated with decreased risk of stroke, myocardial infarction, and heart failure, preventive and interventional programs are limited and not well structured and organized in Jordan. Moreover, population-based preventive programs are lacking in Jordan. In addition, there is scarcity of recent data on hypertension prevalence, awareness, control, and its risk factors. These data are needed for developing prevention and intervention programs to control and manage hypertension.

This study aimed to determine the prevalence, awareness, and control rates of hypertension and their associated factors among Jordanian adults. Moreover, this study aimed to assess the change in these rates between 2009 and 2017.

## 2. Methods

### 2.1. Study Design and Sampling

This survey was conducted among Jordanian adults over a period of four months in the year 2017. The survey methods and procedures are similar to those that had been used in the 2009 survey [8]. A multistage cluster sampling approach with probability proportional to size random selection method was used to ensure adequate coverage of the entire target population. A city/village was selected from each of the 12 governorates of Jordan. The sample of households was chosen in two stages. In the first stage, well-defined geopolitical areas (clusters) were selected from each city/village. At least one cluster was selected from each city/village at random using computer-generated random numbers. The second stage of household selection involved choosing a random sample of households from a list of households in a selected area. The households from each cluster were selected at random using systematic sampling technique. A team of two (a female and a male) visited and invited selected households to report to the health center in that site fasting in a given day after explaining the study for them. Subjects were asked not to take their medications in that day and to bring the medications with them to the health center. Subjects aged ≥18 years were eligible for inclusion in the study. To encourage participation, the team worked on weekends and holidays and provided free transport for those who asked for it. The overall response rate was 78.1%. The total sample participating in the study was 4056 subjects which translates to a margin of error of about 1.3% given a prevalence of 20% and a 95% confidence level.

The study was approved by the Ethical Committee at the National Center for Diabetes, Endocrinology, and Genetics, Amman, Jordan. Informed consent was obtained from each participant. Data were treated with strict confidentiality and used only for scientific purposes.

### 2.2. Data Collection

Trained interviewers administered a comprehensive structured questionnaire specifically prepared for the purpose of the study. Main data obtained included sociodemographic variables, diabetes and other cardiovascular disease risk factors, morbidity, quality of life and health services, and others. Height, weight, waist and hip circumferences, and blood pressure were carried out in a standard way by trained researchers as explained in the 2009 survey [8].

Three blood samples were drawn from a cannula inserted into the antecubital vein and used for the different laboratory measurements. Tubes containing sodium fluoride potassium oxalate were used for glucose measurement. Samples were centrifuged within 1 hour at the survey site and transferred by separate labeled tubes in ice boxes to the central laboratory of the National Center of Diabetes, Endocrinology, and Genetics in Amman, Jordan. All biochemical measurements were carried out by the same team of laboratory technicians using the same method throughout the study period. Fasting plasma glucose was measured by the glucose oxidase method, using a Cobas Analyzer (Roche).

### 2.3. Variable Definitions

Hypertension was defined as average measured blood pressure ≥140 mm Hg systolic and/or 90 mm Hg diastolic, or self-reported use of medications for hypertension [9]. Participants were defined as aware of hypertension if they had hypertension and reported being informed about the diagnosis by a physician. Patients were considered controlled if they had hypertension, on antihypertensive medication and had systolic blood pressure <140 mm Hg and diastolic blood pressure <90 mm Hg. Body mass Index (BMI) was calculated by dividing the weight in kilogram by the height in meters squared. Participants with BMI of 30 kg/m2 or more were considered obese, while those with BMI values that range between 25 kg/m^2^ and <30 kg/m^2^ were considered overweight. Metabolic abnormalities including increased waist circumference, raised fasting plasma glucose, high triglycerides level, and low high density lipoprotein (low HDL) were defined according to the International Diabetes Federation (IDF) definition [10].

### 2.4. Statistical Analysis

Data were entered and analyzed using the Statistical Package for Social Sciences software (SPSS IBM version 20). The raw data file for 2009 was reanalyzed using the same variable definitions to assess the time-trends in hypertension prevalence, awareness, and control. Proportions were used to estimate the prevalence, awareness, and control of hypertension. Overall and age-specific prevalence rates were obtained and reported separately for each gender. To permit comparison between the different surveys and with studies in other countries, we derived age-standardized prevalence rates using the world population as a standard. Ninety-five percent confidence limits were reported standardized rates. Chi-square and crosstabs were used to compare the difference between proportions. Multivariate analysis was conducted using generalized linear mixed models (GLMMs) using a logit link (binary logistic regression) to take into account the clustering of observations. Separate GLMM models were used for assessing the independent effects of individual factors associated with hypertension prevalence, awareness, and control. A p-value of less than 0.05 was considered to be statistically significant.

## 3. Results

### 3.1. Participants' Characteristics

This study included a total of 1193 men and 2863 women. Their aged ranged from 18 to 90 year with a mean (SD) of 43.8 (14.2) year. About 74.6% had increased waist circumference and 42.6% had raised fasting plasma glucose.  Table 1 shows the sociodemographic, anthropometric, and clinical characteristics of participants according to gender. Men and women differed significantly in these characteristics.

### 3.2. Hypertension Prevalence, Awareness and Control

**Figure 1:**
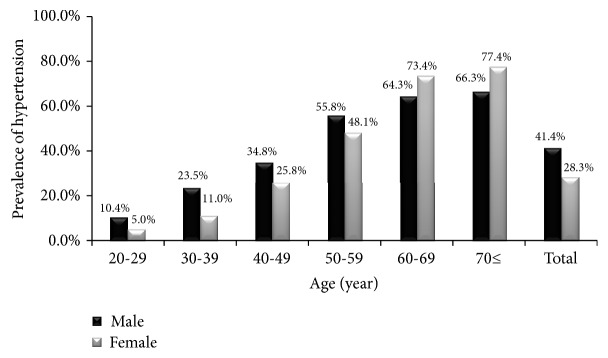
The prevalence of hypertension among Jordanian adults according to age.

**Figure 2:**
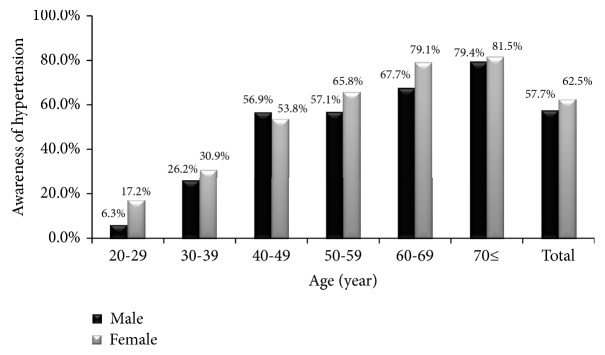
The rate of hypertension awareness among Jordanian adults according to age.

**Figure 3:**
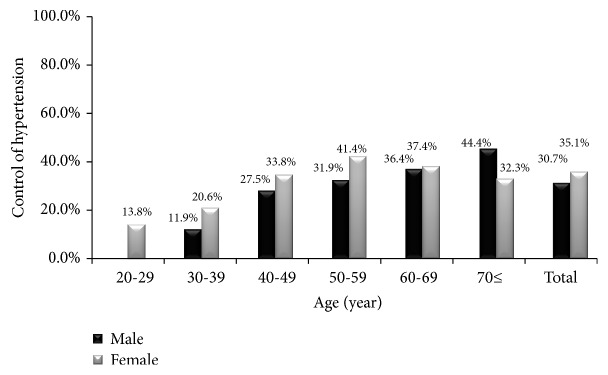
The rate of hypertension control among Jordanian adults according to age.

The crude prevalence of hypertension was 41.4% among men and 28.3% among women. The age-standardized prevalence was 33.8% (95% confidence interval (CI): 31.3%-36.3%) among men and 29.4% (95% CI: 28.0%-30.8%) among women. The prevalence of hypertension increased significantly with increasing age among men and women (Figure 1). Of those with hypertnsion, 57.7% of men and 62.5% of women were aware of hypertension. Only 30.7% of men and 35.1% of women whoe were on antihypertensive medications had their blood pressure controlled. The rates of hypertesnion awareness and control increased significantly with increasing age among men and women (Figures 2 and 3). Tables 2 and 3 show the prevalence, awareness, and control of hypertension among Jordanian men and women according to participants' characteristics.

### 3.3. Change in Hypertension Prevalence, Awareness, and Control between 2009 and 2017

From 2009 to 2017, there was nonsignificant decrease in hypertension prevalence of 2.7% among men and 1.1% among women. This decrease was consistent in men and women, who had an age-standardized hypertension prevalence of 36.5% (33.9-39.2%) and 30.5% (29.2-31.9%) in 2009, respectively. However, the rate of hypertension awareness increased significantly among men from 39.8% in 2009 to 57.7% in 2017 and among women from 51.8% in 2009 to 62.5% in 2017. Similarly, the rate of hypertension control increased from 17.4% to 30.7% among men and from 18.6% to 30.7% among women between 2009 and 2017.

### 3.4. Factors Associated with Hypertension Prevalence, Awareness, and Control

In the multivariate analysis (Table 4), age ≥50 year, increased waist circumference, family history of hypertension, elevated triglycerides level, and increased plasma glucose were significantly associated with increased odds of hypertension among men and women. Married men and women and those with low HDL had higher odds of hypertension. On the other hand, people aged ≥50 years, married people, those with a family history of hypertension, and current smokers were more likely to be aware of hypertension. Of all variables, only age was associated with hypertension control among men. Men aged ≥50 year were twice more likely to have controlled hypertension compared to those aged <50 years. Among women, those aged 50 year and married women were more likely to have controlled hypertension.

## 4. Discussion

This study showed that almost one-third of Jordanian adults had hypertension. The age-standardized prevalence of hypertension was 33.8% among men and 29.4% among women. The prevalence of hypertension varies widely across the Arab countries. A systematic review of 13 studies from 10 Arab countries reported an overall estimated prevalence of hypertension of 29.5% [7]. Another systematic review reported an overall worldwide prevalence of 26% in the adult population [11]. The differences in the prevalence rates between countries might be explained differences among studied populations, sampling methods, study settings, and timeframes of the studies. The high rate of obesity and physical inactivity and high salt and fat intake in Jordan might explain the high prevalence of hypertension in Jordan as well as other Arab countries.

Consistent with the findings of many studies in the world [12, 13], including studies in Arab countries [8, 14], the rate of hypertension was found to increase by age in both genders. In our study, the prevalence was significantly higher among men than that in women. This finding is consistent with the findings of some studies in Arab countries [14–16]. However, other studies found that hypertension is more common in women [17–19]. No significant gender difference in the rate of hypertension was reported in other studies [20, 21].

Our study showed that 57.7% of men and 62.5% of women were aware of hypertension. The systematic review of studies in Arab countries showed that the awareness of hypertension varied from 18% to 79.8% with an overall rate of 46% [7]. A systematic review of studies worldwide showed that the awareness rates ranges from 25 to 75% [11]. Almost half to two-thirds of patients with hypertension in developed countries were aware of their diagnosis [11]. The rate of hypertension awareness in Jordan increased significantly from 39.8% in 2009 to 57.7% in 2017 among men and from 51.8% in 2009 to 62.5% in 2017 among women. The increased awareness from 2009 to 2017 might be explained by the better access to healthcare services in Jordan in the last 10 years.

Almost one-third (30.7% of men and 35.1% of women) of Jordanian adults on antihypertensive medications had controlled hypertension. The rate of hypertension control in Arab countries varied from 8% to 44% [15–20]. The low control rate was also seen in USA and European countries [12]. The poor hypertension control in Jordan might be explained by inadequate management of hypertension, not using evidence based practices in management of hypertension, and poor adherence to medication. On the other hand, the rate of hypertension control increased from 17.4% to 30.7% among men and from 18.6% to 30.7% among women between 2009 and 2017. The improved level of peoples' awareness and improved access to health services over the last few years might explain the increase in the rate of hypertension control.

The multivariate analysis showed that patients aged ≥50 years were more likely to have hypertension, to be of aware of the diseases and to have better control compared to younger patients. The higher rate of hypertension awareness and control among older patients might be explained by that old patients have more frequent visits to health facilities because of other comorbidities and have a higher chance to be informed of their blood pressure and to be prescribed medications to control hypertension. Family history of hypertension was also associated with higher odds of hypertension and awareness of hypertension. Patients with family history of hypertension might learn from their families' experiences and be more likely to attend the health center to check their blood pressure. Increased waist circumference, diabetes, and low HDL were all associated significantly with hypertension. These variables are well-known risk factors for cardiovascular diseases and had been show to cluster in a form of metabolic syndrome.

In conclusion, almost one-third of Jordanian adults had hypertension. Of those with hypertension, more than half of men and about two-thirds of women were aware of hypertension. Only one-third of those who were on antihypertensive medications had controlled blood pressure indicating gaps in the management of hypertension in this country. Interventions that target modifiable risk factors of hypertension, might decrease blood pressure, and even prevent the development of hypertension should be implemnted. Evidence-based prevention and management recommendations and guidelines including lifestyle modifications need to be adopted in Jordan.

## Figures and Tables

**Table 1:** The sociodemographic, anthropometric, and clinical characteristics of participants according to gender.

	Men		Women		Total	P-value
	n	%	n	%	N	
Age (year)						<0.001
<50	648	54.4	1966	68.8	2614	
≥50	543	45.6	890	31.2	1433	
Marital status						<0.001
Single	144	12.1	462	16.1	606	
Married	1049	87.9	2401	83.9	3450	
Region						<0.001
North	390	32.7	922	32.2	1312	
Middle	471	39.5	1295	45.2	1766	
South	332	27.8	646	22.6	978	
Smoking status						<0.001
None smoker	592	49.6	2628	91.8	3220	
Past smoker	206	17.3	46	1.6	252	
Current smoker	395	33.1	189	6.6	584	
Family history of hypertension	665	56.2	1734	61.1	2399	<0.001
Diagnosed with hypertension	336	28.5	614	21.6	950	<0.001
Body mass index (Kg/m^2^)						<0.001
Normal	264	22.7	639	22.7	903	
Overweight	479	41.2	822	29.2	1301	
Obesity	419	36.1	1357	48.2	1776	
Increased waist circumference	797	67.3	2198	77.7	2995	<0.001
Diabetes mellitus*∗*	658	55.2	1069	37.3	1727	<0.001
High triglycerides level	647	54.2	1036	36.2	1683	<0.001
Low HDL	732	61.4	1659	57.9	2391	<0.001

*∗*Fasting blood sugar >100mg/dl or diagnosed with diabetes or on diabetes medication.

**Table 2:** The prevalence, awareness, and control of hypertension among men in Jordan according to participants' characteristics.

	Hypertension			Awareness of hypertension			Control of hypertension		
	n	%	p-value	n	%	p-value	n	%	p-value
Age (year)			<0.001			<0.001			.001
<50	167	25.9		74	44.3		35	21.0	
≥50	325	60.1		210	64.6		116	35.7	
Marital status			<0.001			.001			.053
Single	14	9.9		2	14.3		1	7.1	
Married	478	45.7		282	59.0		150	31.4	
Smoking status			<0.001			<0.001			.011
None smoker	258	43.8		124	48.1		65	25.2	
Past smoker	103	50.0		79	76.7		42	40.8	
Current smoker	131	33.3		81	61.8		44	33.6	
Region			<0.001			.340			.864
North	167	43.0		104	62.3		51	30.5	
Middle	178	37.9		99	55.6		57	32.0	
South	147	44.5		81	55.1		43	29.3	
Family history of hypertension			.058						.217
Yes	292	44.0		188	64.4		96	32.9	
No	199	38.5		96	48.2		55	27.6	
Body mass index (Kg/m^2^)			<0.001			.330			.874
Normal	59	22.3		34	57.6		20	33.9	
Overweight	198	41.3		109	55.1		61	30.8	
Obesity	217	51.8		135	62.2		66	30.4	
Wasit circumference			<0.001			.076			.394
Normal	93	24.0		46	49.5		25	26.9	
Increased	398	49.9		237	59.5		125	31.4	
Diabetes			<0.001			.016			
No	142	26.7		70	49.3				.602
Yes	350	53.4		214	61.1		46	32.4	
Triglycerides level			<0.001			.602			
Normal	178	32.8		100	56.2		55	30.9	
High	314	48.6		184	58.6		96	30.6	
HDL			.014			.100			.978
Normal	169	37.0		89	52.7		52	30.8	
Low	323	44.2		195	60.4		99	30.7	

**Table 3:** The prevalence, awareness, and control of hypertension among women in Jordan according to participants' characteristics.

	Hypertension			Awareness of hypertension			Control of hypertension		
	n	%	p-value	n	%	p-value	n	%	p-value
Age (year)			<0.001			<0.001			.004
<50	292	15.0		131	44.9		84	28.8	
≥50	510	58.0		370	72.5		198	38.8	
Marital status						.000			.001
Single	53	11.6		11	20.8		7	13.2	
Married	750	31.5		491	65.5		275	36.7	
Smoking status			.160			.001			.003
None smoker	725	27.9		438	60.4		241	33.2	
Past smoker	17	37.0		14	82.4		8	47.1	
Current smoker	61	32.6		50	82.0		33	54.1	
Region			0.431			.794			.532
North	256	28.0		156	60.9		87	34.0	
Middle	352	27.2		224	63.6		120	34.1	
South	195	31.1		122	62.6		75	38.5	
Family history of hypertension			<0.001			<0.001			.005
Yes	567	33.0		387	68.3		217	38.3	
No	234	21.4		115	49.1		65	27.8	
Body mass index (Kg/m^2^)			<0.001			.160			.335
Normal	48	7.5		24	50.0		12	25.0	
Overweight	163	19.9		106	65.0		58	35.6	
Obesity	578	42.7		363	62.8		205	35.5	
Wasit circumference			<0.001			.005			.058
Normal	35	5.6		14	40.0		7	20.0	
Increased	763	34.7		484	63.4		272	35.6	
Triglycerides level			<0.001			.000			.940
Normal	365	20.3		204	55.9		118	32.3	
High	438	42.4		298	68.0		164	37.4	
HDL			<0.001			.079			.393
Normal	240	20.3		139	57.9		79	32.9	
Low	563	34.1		363	64.5		203	36.1	
Diabetes			<0.001			<0.001			
No	302	17.0		147	48.7				.032
Yes	501	47.1		355	70.9		92	30.5	

**Table 4:** The multivariate analysis of factors associated hypertension prevalence, awareness, and control.

	*Men*		*Women*	
	OR (95% Confidence interval)	P-value	OR (95% Confidence interval)	P-value
*Prevalence of Hypertension*				
Age (≥50 vs. <50)	3.3 (2.5, 4.4)	<0.001	5.0 (4.1, 62)	<0.001
Increased waist circumference	2.0 (1.5, 2.8)	<0.001	3.7 (2.5, 5.5)	<0.001
Marital status (married vs. single)	2.2 (1.2, 4.1)	0.015		
Family history of hypertension	1.5 (1.1, 2.0)	0.004	1.9 (1.6, 2.4)	<0.001
Elevated triglycerides level	1.6 (1.2, 2.1)	0.001	1.4 (1.2, 1.8)	0.001
Diabetes	1.7 (1.3, 2.2)	<0.001	1.4 (1.1,1.7)	<0.001
Low HDL	1.4 (1.1, 1.7)	0.004		
*Awareness of hypertension*				
Family history of hypertension	2.8 (1.8, 4.4)	<0.001	3.2 (2.2, 4.6)	<0.001
Age (≥50 vs. <50)	2.5 (1.6, 3.9)	<0.001	3.4 (2.5, 4.8)	<0.001
Marital status (married vs. single)	5.6 (1.2, 26.7)	0.032	6.3 (3.0,13.0)	<0.001
Smoking				
None smoker	1		1	
Past smoker	3.4 (2.0, 6.2)	<0.001	3.2 (0.8,13.6)	0.114
Current smoker	1.8 (1.1, 3.0)	0.009	2.9 (1.4, 6.1)	0.004
*Control of hypertension*				
Age (≥50 vs. <50)	1.8 (1.5, 2.3)	<0.001	3.0 (2.2, 4.1)	<0.001
Marital status (married vs. single)			5.9 (2.9, 11.9)	<0.001

## Data Availability

The data used to support the findings of this study are available from the corresponding author upon request.
